# Supplementary material for: RanBP1: A Potential Therapeutic Target for Cancer Stem Cells in Lung Cancer and Glioma
Source: Int J Mol Sci. 2023 Apr 6;24(7):6855. doi: 10.3390/ijms24076855 (PMC10095367; doi:10.3390/ijms24076855)

Supplementary figure legends

Figure S1. Uncropped western blot images. (A) Uncropped western blot images of Figure 1B. (B) Uncropped western blot images of Figure 2D. (C) Uncropped western blot images of Figure 2E. (D) Uncropped western blot images of Figure 3A. (E) Uncropped western blot images of Figure 3B. (F) Uncropped western blot images of Figure 4E. (G) Uncropped western blot images of Figure 4F. (H) Uncropped western blot images of Figure 4G. (I) Uncropped western blot images of Figure 4F. (J) Uncropped western blot images of Figure 5A. (K) Uncropped western blot images of Figure 5D.

Supplementary Material 1.

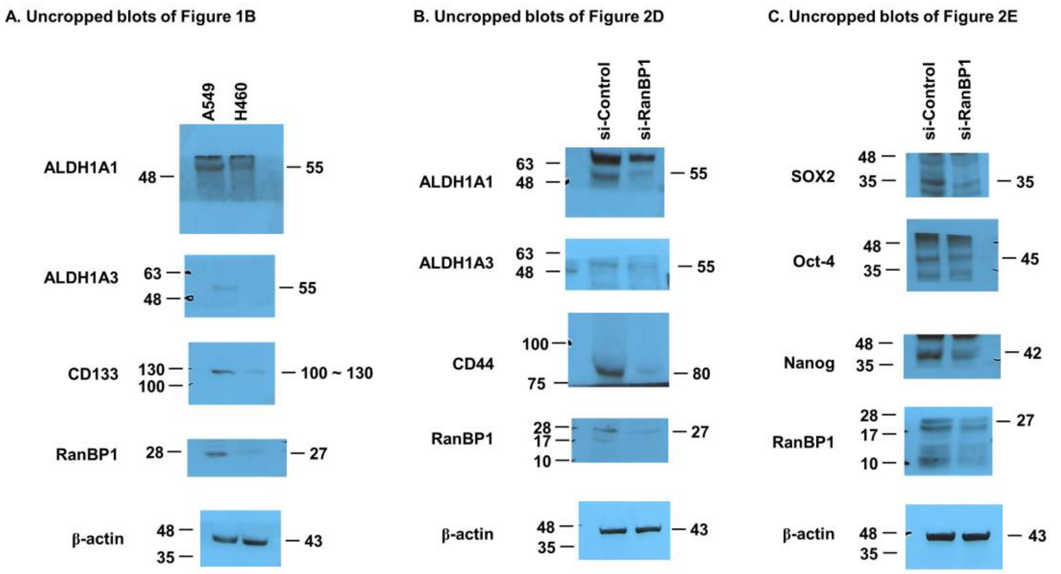

Supplementary Material 1.

D. Uncropped blots of Figure 3A

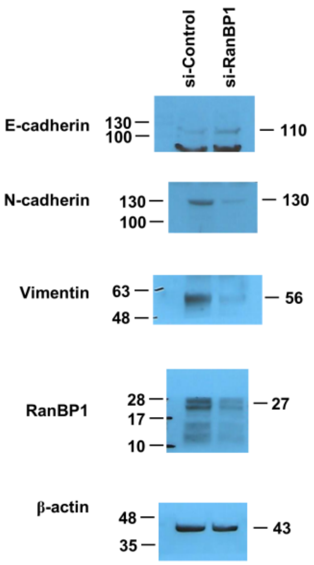

E. Uncropped blots of Figure 3B

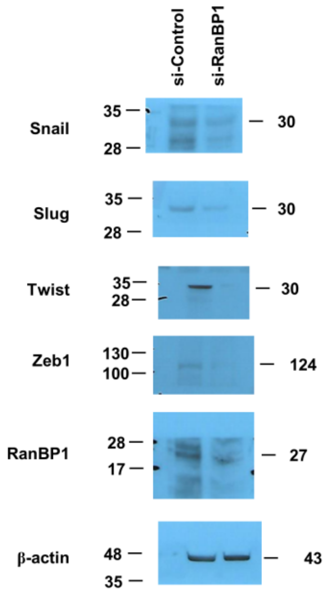

Supplementary Material 1.

F. Uncropped blots of Figure 4E   G. Uncropped blots of Figure 4F   H. Uncropped blots of Figure 4G

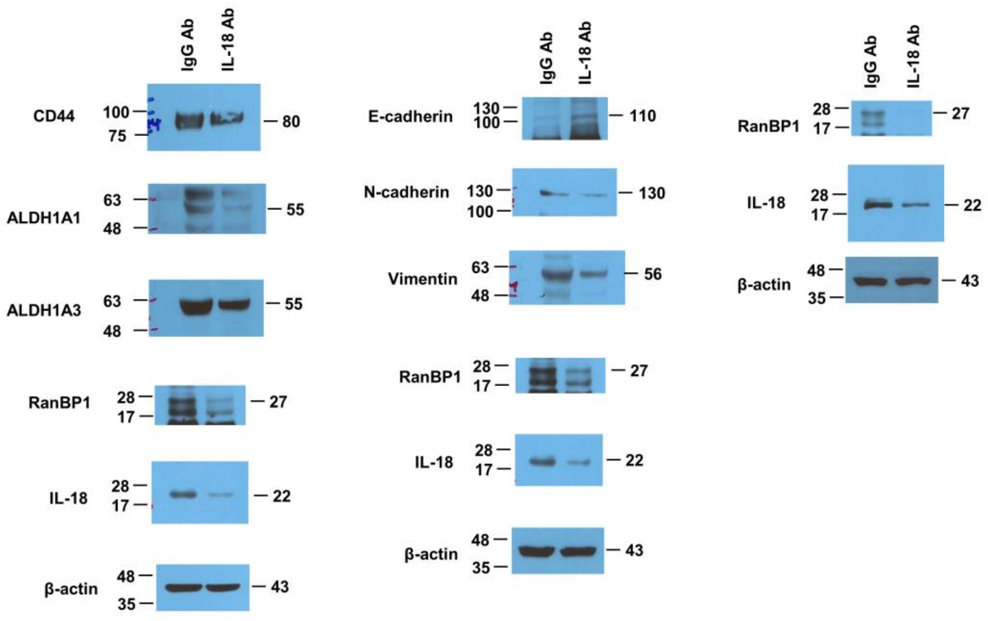

Supplementary Material 1.

I. Uncropped blots of Figure 5A   J. Uncropped blots of Figure 5D

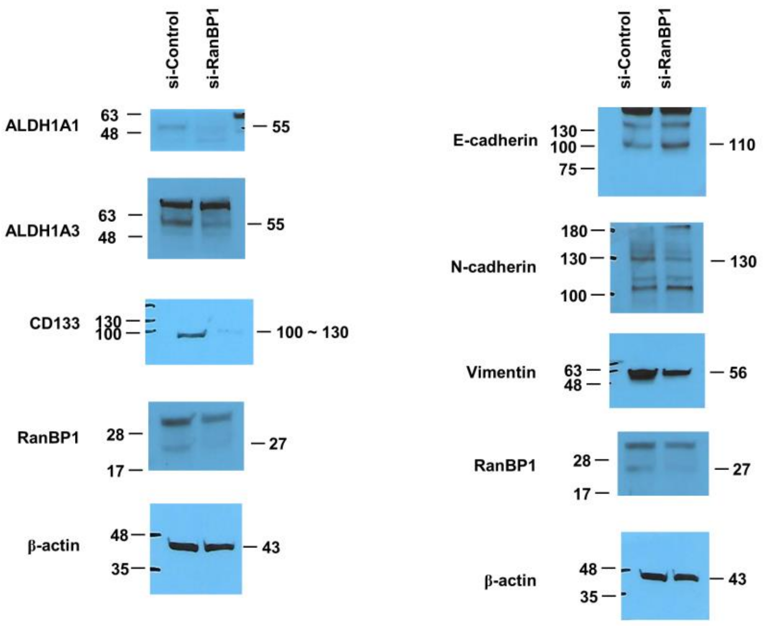

Supplement: Supplementary file 1 [file ijms-24-06855-s001.zip › ijms-2278422-supplementary.pdf]
